# Feasibility of an Electronic Patient-Reported Outcome Tool for Screening Distress and Supportive Care Needs of Adolescents and Young Adults with Cancer

**DOI:** 10.1089/jayao.2023.0014

**Published:** 2024-02-09

**Authors:** Takatoshi Hirayama, Hiroto Ishiki, Yuko Yanai, Saki Horiguchi, Akiko Sugisawa, Jun Sato, Ryugo Kojima, Kaori Sato, Tomoko Mizuta, Rebekah Kojima, Ryoko Udagawa, Yuki Kojima, Eriko Satomi

**Affiliations:** ^1^ Department of Psycho-Oncology and National Cancer Center Hospital, Chuo-ku, Tokyo, Japan.; ^2^ Department of Palliative Medicine, National Cancer Center Hospital, Chuo-ku, Tokyo, Japan.; ^3^ Department of Nursing, National Cancer Center Hospital, Chuo-ku, Tokyo, Japan.; ^4^ Department of Pharmacy, and National Cancer Center Hospital, Chuo-ku, Tokyo, Japan.; ^5^ Department of Medical Oncology, National Cancer Center Hospital, Chuo-ku, Tokyo, Japan.

**Keywords:** adolescent and young adult, electronic patient-reported outcome, screening, distress, supportive care needs, multidisciplinary approach

## Abstract

**Purpose:**

Although adolescent and young adult (AYA) cancer patients are digital natives and have high digital communication needs, previous studies of screening tools for AYAs have primarily used paper when measuring patient-reported outcomes (PROs). There are no reports on using an electronic PRO (ePRO) screening tool with AYAs. This study evaluated the feasibility of such a tool in clinical settings, and assessed the prevalence of AYAs' distress and supportive care needs.

**Methods:**

An ePRO tool based on the Distress Thermometer and Problem List (DTPL)-Japanese (DTPL-J) version for AYAs was implemented in a clinical setting for 3 months. To determine the prevalence of distress and need for supportive care, descriptive statistics were calculated for participant characteristics, selected items, and Distress Thermometer (DT) scores. Response rates, referral rates to an attending physician and other experts, and time required to complete PRO tools were assessed to evaluate feasibility.

**Results:**

From February to April 2022, 244 (93.8%) of 260 AYAs completed the ePRO tool based on the DTPL-J for AYAs. Based on a DT cutoff of ≥5, 65 of 244 patients (26.6%) had high distress. Worry (*n* = 81, 33.2%) was the most frequently selected item. Primary nurses referred 85 (32.7%) patients to an attending physician or other experts. The referral rate resulting from ePRO screening was significantly higher than that after PRO screening (*χ*^2^(1) = 17.99, *p* < 0.001). The average response time did not differ significantly between ePRO and PRO screening (*p* = 0.252).

**Conclusion:**

This study suggests the feasibility of an ePRO tool based on the DTPL-J for AYAs.

## Introduction

Adolescent and young adult (AYA) cancer patients have various needs, such as those related to friendship,^1^ employment,^2^ education,^3^ health behaviors,^4^ sexuality,^5,6^ and social and family issues.^7^ However, many of these needs are unmet.^8,9^ Over 70% of AYAs in Japan had an unmet need for supportive care,^10^ and this was found to be associated with distress^11^ and decreased quality of life.^12^

To meet the needs of AYAs in terms of distress and supportive care, the Distress Thermometer and Problem List (DTPL) was developed by the National Comprehensive Cancer Network (NCCN)^13^ and has been used in various countries. In Singapore, the DTPL was useful in the identification of clinically relevant psychological distress in AYAs during the initial phase of their journey with cancer.^14^ In Australia, an AYA-specific screening tool based on the DTPL helped clinicians support AYAs' psychosocial coping in active treatment and encouraged healthy survivorship after treatment.^15^ The tool has already been validated in a multinational study in mainly English-speaking countries.^16^

In Australia, psychosocial support is being implemented as a national project using the tool.^17^ In Japan, a Japanese version of the DTPL (DTPL-J) for AYAs was developed, and its feasibility, discriminant validity, and test–retest reliability were suggested by our research team.^18^ In addition, the feasibility and preliminary effectiveness of a psychosocial support program based on the DTPL-J for AYAs were reported by a multicenter study.^19^

A systematic review of 24 controlled trials found that the routine use of patient-reported outcomes (PROs) increases consultations through improved physician–patient communication, symptom identification and control, and patient satisfaction.^20^ A review of PRO labeling for oncology drugs approved by the Food and Drug Administration (FDA) and the European Medicines Agency (EMA) suggested that, at a minimum, consideration should be given to the use of PRO measures that assess patient-centered proximate concepts of core disease symptoms, treatment-related symptoms, and impacts on functioning.^21^

Traditional paper PROs seem to be gradually replaced by electronic PROs (ePROs). In the field of oncology, ePROs were found to be more effective than paper-based PROs due to reduced data entry errors, improved patient disclosure of sensitive information, prompt access to data, and the ability to provide trigger alerts and messages.^20^ Oncologists recognized the potential benefits of implementing ePRO systems in real-world practice, such as improved patient-centered care and clinical efficiency.^22^ A previous study reported that the incorporation of PROs into the routine care of cancer patients was associated with an increase in survival over usual care,^23^ and that self-report of symptoms during cancer care was associated with clinical utility.^24^

With regard to the implementation of ePRO systems, the International Society for Pharmacoeconomics and Outcomes Research (ISPOR) ePRO good research practices task force released recommendations on the necessary evidence to support measurement equivalence between ePRO and PRO measures,^25^ and one study discussed key methodological issues to be concerned with in planning of usability testing of ePRO systems.^26^

However, previous studies of screening tools for AYAs have primarily used paper-based PRO tools. Although AYAs are digital natives and have high digital communication needs,^27^ the feasibility of implementing an ePRO screening tool in the AYA population is not reported.

The objective of this study was to assess the feasibility of an ePRO tool for screening and determining the prevalence of distress and supportive care needs among AYAs.

## Methods

### Study design

This retrospective medical records-based study was designed to assess the feasibility of an ePRO tool based on the DTPL-J for AYAs and to report the prevalence of distress and supportive care needs in this population. This ePRO tool was implemented in a clinical setting for 3 months at the National Cancer Center Hospital (NCCH), Japan. The eligibility criteria for AYAs were as follows: (1) age between 15 and 39 years, (2) histological diagnosis of malignant neoplasm between the ages of 15 and 39 years, and (3) hospitalization at NCCH at any time from February to April 2022.

We compared outcomes in this sample with those of individuals who completed a paper-based PRO tool based on the DTPL-J for AYAs in our previous feasibility study;^18^ data selected from that study were originally obtained from February to April 2020, corresponding to the same time of year as that used in this study.

This study was approved by our institutional review board (IRB number: 2019-215). Because of the retrospective design, it was only required that opt-out information be published on the NCCH website.

### ePRO tool based on the DTPL-J for AYAs

The DTPL-J for AYAs consists of the Distress Thermometer (DT) and a problem list of 49 items in five categories: physical problems, family problems, practical problems, emotional problems, and spiritual or religious concerns.^18^ Physical problems comprise items related to one's appearance (the way that you look), bathing or dressing, breathing, changes in urination, constipation, diarrhea, eating, indigestion, fatigue, feeling swollen, fever, daily activity, memory or concentration, mouth sores, nausea, nasal dryness or congestion, pain, sexual issues, dryness or itchiness of skin, sleep, tingling of hands or feet, and use of nonprescription medicine.

Family problems consist of items associated with interaction with parents, dealing with children, dealing with partner, interaction with other family members, ability to have children, and physical or mental health of family members. Practical problems are items related to money (medical expenses, living expenses, and insurance), transportation (visiting hospitals and commute to school or work), work or school, treatment options, information about illness or treatment, someone to talk to or consultation environment, important schedule or events, interaction with medical staff, interaction with people other than your family members, hospitalization life, child care, and housing. Emotional problems consist of the items pertaining to depression, anxiety, irritation, fear, nervousness, sadness, worry, and loss of interest in usual activities.

A tablet personal computer was implemented for the ePRO tool in this study. Regarding the change from paper to digital format, the paper-and-pencil format (210 × 297 mm) was simply rescaled to a screen text format (208 × 287 mm) without greatly decreasing font size or modifying item content or response options. This is considered a small change based on the ISPOR ePRO good research practices task force report.^25^

Electronic data processing made it possible to use the ePRO data immediately after the medical encounter, and the data were automatically linked to the patients' electronic medical record.

### Procedures

The ePRO tool was used by nurses to assess patients on admission to NCCH. Primary support was provided by nurses. After screening patients to assess their needs, nurses informed them of the support they needed. In addition, with patient consent, the information collected was shared with the primary care team. Early expert support was provided to patients requiring intervention. The main occupations of the experts providing secondary support were attending physicians, appearance care staff, certified nurse specialists, fertility consultation nurses, hospital play staff, medical social workers, and psycho-oncology department staff.

Appearance care staff used medical, cosmetic, and psychosocial support to provide care that complements changes in appearance and reduced the distress of cancer patients caused by changes in appearance. Psycho-oncology department was composed of psychiatrists and psychotherapists who supported patients in pairs.

Patients' medical records were investigated retrospectively. Some patients had multiple screenings because a screening was done separately at each hospitalization. Response rates, referral rates to an attending physician and other experts, and time required for ePRO tool input were evaluated to assess the feasibility of the first screening. Sample size was not determined in advance because this was a retrospective study to assess the feasibility of the ePRO tool based on the DTPL-J for AYAs.

### Analysis

To determine the prevalence of distress and need for supportive care, descriptive statistics were calculated for participant characteristics, items selected from the problem list, and the DT score. Associations between these parameters were examined using the chi-square test. The correlation between the DT score and the number of selected items was determined by correlation analysis. A DT score ≥5 was defined as high distress.^9^

Response rates, referral rates to an attending physician and other experts, and response times were evaluated to assess feasibility. We preliminarily defined the ePRO tool as feasible if the response rate was ≥91.6%, since this was the response rate achieved with the paper-based DTPL-J for AYAs in our previous study.^18^ The response rate was defined as the percentage of AYAs who completed the ePRO tool after it was first administered from February to April 2022. Using the chi-square test, the referral rate to an attending physician and other experts was compared between ePRO screening in this study and PRO screening in our previous study.^18^

Using the unpaired *t*-test, the time required to complete PRO screening was compared between 25 random AYAs (10.2%) who were administered the ePRO tool in this study and 23 random AYAs (10.0%) who were provided with the paper-based PRO tool in our previous study.^18^ A two-sided *p*-value <0.05 was regarded as significant. Analyses were conducted using IBM SPSS Statistics version 27 (IBM Corp, Armonk, NY, USA).

## Results

### Response rate

**FIG. 1.**
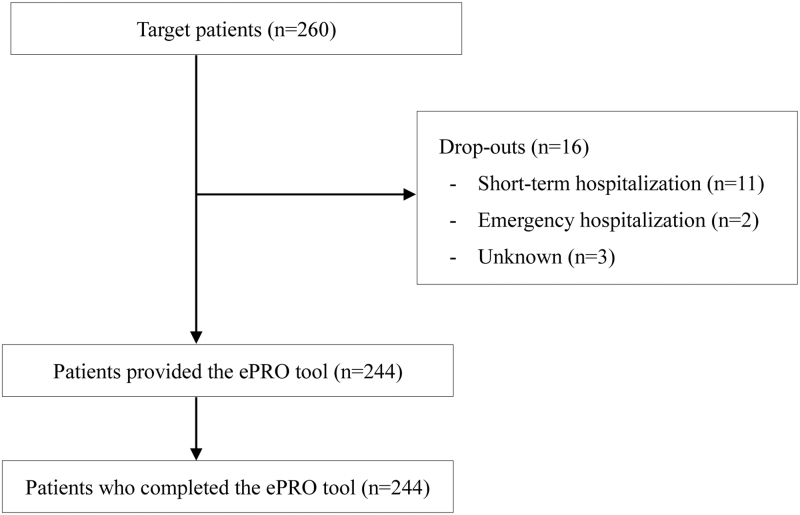
Patient flow.

Patient flow is shown in Figure 1. Of 260 AYAs, 16 did not complete the ePRO tool at least once between February and April 2022, for the following reasons: emergency (*n* = 11) or short-term hospitalization (*n* = 2) and unknown (*n* = 3). Thus 244 (93.8%) AYAs completed the ePRO tool during the specified period.

Respondents' demographic characteristics are given in Table 1. There were 124 females and 120 males, with an average age of 25.0 years. Primary malignancy sites included bone and soft tissue (*n* = 113, 46.3%), hematological (*n* = 26, 10.7%), colorectal (*n* = 17, 7.0%), gynecological (*n* = 15, 6.1%), breast (*n* = 13, 5.4%), testicle (*n* = 13, 5.4%), and others (*n* = 47, 19.1%). The most frequent stage at diagnosis was stage IV (*n* = 93, 38.1%), followed by stage III (*n* = 37, 15.2%). The most frequent treatment setting was curative (*n* = 149, 61.1%).

**Table 1.** Respondents' Demographic Characteristics (*N* = 244)

	No. of patients	Proportion of patients (%)
Age, years (mean 25.0 ± 7.6)		
15–19	75	30.8
20–24	55	22.5
25–29	39	16.0
30–34	33	13.5
35–39	42	17.2
Gender		
Female	124	50.8
Male	120	49.2
Educational status		
High school graduate	61	25.0
Junior high school graduate	59	24.2
College graduate	24	9.8
Vocational school graduate	4	1.6
Unknown	96	39.4
Social status		
Employed	102	41.8
Student	99	40.6
Unemployed	43	17.6
Cancer type		
Bone and soft tissue tumor	113	46.3
Hematological cancer	26	10.7
Colorectal cancer	17	7.0
Gynecological cancer	15	6.1
Breast cancer	13	5.4
Testicular cancer	13	5.4
Germ cell tumor	12	4.9
Brain tumor	10	4.1
Retroperitoneal tumor	7	2.9
Gastric cancer	4	1.6
Paraganglioma	3	1.2
Esophageal cancer	2	0.8
Pancreatic cancer	2	0.8
Renal cancer	2	0.8
Peritoneal cancer	2	0.8
Other	3	1.2
Disease stage		
0–II	28	11.4
III	37	15.2
IV	93	38.1
Other	26	10.7
(Hematological cancer)		
Unknown	60	24.6
Treatment setting		
Curative	149	61.1
Palliative	80	32.8
Pre-treatment	15	6.1
Cancer treatment history^a^		
Chemotherapy	191	78.3
Surgery	108	44.3
Radiation	66	27.0
Transplantation	6	2.5
None	39	16.0
Cancer treatment in progress^a^		
Chemotherapy	155	63.5
Surgery	35	14.3
Radiation	15	6.1
Transplantation	7	2.9
None	49	20.1
Has spouse or partner		
No	176	72.1
Yes	68	27.9
Parent		
No	218	89.3
Yes	26	10.7
Living situation		
Living with someone else	219	89.8
Living alone	25	10.2

^a^ Multiple responses allowed for each patient.

### DT score and supportive care needs

As defined by a DT ≥5, 65 of 244 patients (26.6%) had high distress. In the high-distress group (*n* = 65), 64 patients (98.5%) selected at one or more items from the problem list. In the low-distress group (*n* = 179), 135 patients (75.4%) selected at one or more items (*χ*^2^(1) = 16.83, *p* < 0.001).

All items in the problem list were selected two or more times. The average number of items selected was 6.6. Worry (*n* = 81, 33.2%) was the most frequently selected item. The DT score was positively correlated with the number of items selected (*r* = 0.589, *p* < 0.001).

### Referral rates to an attending physician or other experts

Among the patients who responded, 199 (81.6%) selected at one or more items and 85 (32.7%) were referred to an attending physician or other experts by their nurse (Table 2). This referral rate was significantly higher than that based on PRO screening (45 of 251, 18%) (*χ*^2^ (1) = 17.99, *p* < 0.001).

**Table 2.** Items Checked by Patients and Referrals to an Attending Physician and Multidisciplinary Experts

			Primary support		Secondary support													
			Nurse		Attending physician		Appearance care staff		CNS		Fertility consultation nurse		HPS		MSW		Psycho-oncology department staff	
	*n*	%	*n*	%	*n*	%	*n*	%	*n*	%	*n*	%	*n*	%	*n*	%	*n*	%
Physical problem																		
Appearance (the way that you look)	50	20.5	23	46			10	20										
Items not related to appearance	156	63.9	56	35.9	12	7.7	4	2.6	1	0.6							5	3.2
Fatigue	61	25.0																
Pain	59	24.2																
Eating	49	20.1																
Sleep	44	18.0																
Tingling of hands or feet	39	16.0																
Constipation	38	15.6																
Dryness or itchiness of skin	35	14.3																
Daily activity	34	13.9																
Nasal dryness or congestion	31	12.7																
Nausea	29	11.9																
Bathing or dressing	26	10.7																
Diarrhea	21	8.6																
Feeling swollen	21	8.6																
Changes in urination	21	8.6																
Fevers	19	7.8																
Indigestion	18	7.4																
Breathing	16	6.6																
Memory or concentration	15	6.1																
Mouth sores	12	4.9																
Sexual issues	9	3.7																
Use of nonprescription medicine	8	3.3																
Family problem																		
Ability to have children	27	11.1	14	51.9							5	18.5						
Ability to have children Items other than ability to have children	54	22.1	28	51.9									4	7.4				
Physical or mental health of family members	46	18.9																
Interaction with parents	21	8.6																
Dealing with partner	13	5.3																
Interaction with other family members	12	4.9																
Dealing with children	10	4.1																
Practical problems																		
Money matters and someone to talk to or consultation environment	70	28.7	26	37.1											3	4.3		
Money matters (medical expenses, living expenses, insurance)	63	25.8																
Someone to talk to or consultation environment	16	6.6																
Items other than money matters and someone to talk to or consultation environment	117	48.0	46	39.3			5	4.3	1	0.9			1	0.9	1	0.9		
Work or school	68	27.9																
Information about illness or treatment	55	22.5																
Hospitalization life	51	20.9																
Treatment options	41	16.8																
Transportation (visiting hospitals, commute to school or work)	32	13.1																
Important schedule or events	32	13.1																
Interaction with people other than your family members	27	11.1																
Interaction with medical staff	24	9.8																
Housing	19	7.8																
Child care	15	6.1																
Emotional problems	115	47.1	54	47			6	5.2	3	2.6			6	5.2			18	15.7
Worry	81	33.2																
Anxiety	74	30.3																
Depression	53	21.7																
Nervousness	47	19.3																
Fear	44	18.0																
Sadness	36	14.8																
Irritation	24	9.8																
Loss of interest in usual activities	16	6.6																
Spiritual or religious concerns	2	0.8	0	0														

CNS, certified nurse specialist; HPS, hospital play staff; MSW, medical social worker.

### PRO response times

The average response time with the ePRO tool was 151 seconds (standard deviation [SD] 98), compared with 122 seconds (SD 77) for the paper tool, indicating no significant difference (*p* = 0.252).^18^

## Discussion

In this study, we assessed the feasibility of administering an ePRO tool based on the DTPL-J for AYAs in the clinical setting.

The response rate was 93.8%, exceeding our preliminary feasibility of 91.6%. The referral rate to multidisciplinary experts was 32.7%, which was significantly higher than the 18% resulting from use of the paper-based PRO tool. This may reflect the fact that the ePRO data were immediately available digitally and were automatically linked to patients' electronic medical records. Thus, a multidisciplinary approach is required to manage AYAs,^8,9^ could be more easily facilitated by the ePRO tool than by the paper-based tool. Screening tools for the initiation of psychosocial and comprehensive care for AYAs were reported to be useful in previous studies.^14,15^ As such, the ePRO tool could improve the effectiveness of AYA support teams and ensure consistent support for AYAs.

In general, ePRO tools for AYAs are considered to be associated with faster response times than paper-based tools, although this was not the case in this study (*p* = 0.252). Although the time to complete the ePRO was measured automatically, the time to fill out the paper sheet was measured by the person in charge, and this person's presence may have increased the pressure felt by the AYAs. Although the response times for the two types of tools were similar, the response rate and the referral rate to multidisciplinary experts were higher for the ePRO tool, suggesting its greater feasibility.

Although the percentage of AYAs with high distress was 26.6% in this study, a previous study that used the same DT cutoff of ≥5 reported that 42% of AYAs experienced distress within 3 months of diagnosis.^16^ Our study possibly has enrolled AYAs who were diagnosed well before ePRO screening because the time since diagnosis was not included in the eligibility criteria. Since distress in AYAs decreases with time after diagnosis,^14^ the prevalence of distress in this study was possibly less than in previous studies.

In addition, several patients in this study who required supportive care expressed significant distress, which is in line with the findings of a previous study.^13^ Barriers experienced by AYAs in accessing psychosocial care are multifactorial. Implementing standardized referral systems and repeatedly introducing psychosocial care are imperative.^28^ However, screening with the ePRO tool and intervening more quickly could also help alleviate AYA's distress and ensure continuous and comprehensive supportive care.

Worry (*n* = 81, 33.2%) was the most frequently selected item in this study, and was also one of the most frequently endorsed in previous studies.^16,18^ AYAs have various unmet needs^1–7^ and, therefore, might experience a variety of related worries. Many patients who participated in this study had cancer types associated with specific stressors. Bone and soft tissue cancers have been reported to cause worries about chemotherapy potentially affecting fertility.^29,30^ Hematological cancer patients often require long and, therefore, disruptive treatment interventions that may increase likelihood of adverse events and late effects.

In colorectal cancer, all 14 studies in a systematic review indicated that ostomy-related problems negatively impacted perceived quality of life.^31^ Gynecological and testicular cancers directly affect fertility. Breast cancer patients were shown to experience psychological distress. In particular, they experienced fear of cancer recurrence.^32^

There are several limitations to this study. First, the clinical use of the ePRO tool was implemented at a single cancer center. Owing to the robust nature of the NCCH AYAs support system, our findings may not be applicable to other settings.^33–35^ The AYA support team was composed of multidisciplinary experts who met routinely to discuss the support status of AYAs. Japan has few AYAs at each hospital, and the primary cancer site varies, resulting in each hospital having a shortage of staff and inadequate resources for experts.^36^

More studies are required to assess whether an ePRO tool based on the DTPL-J for AYAs is also applicable in other hospitals. Second, the presence of selection bias may mean that although AYAs in this study experienced distress and had supportive care needs, this might not necessarily be true of AYAs throughout Japan. Third, although this study suggested the feasibility of an ePRO tool based on the DTPL-J for AYAs, it did not prove that electronic and paper-based PRO measures yield equivalent measurements. As the next step, we need to assess their measurement equivalence based on existing recommendations.^25^ Finally, this study did not assess the effectiveness of the ePRO tool in evaluating distress relief, and more studies need to assess this issue by comparing DT scores before and after ePRO screening.

Despite its limitations, this study suggests the feasibility of an ePRO tool based on the DTPL-J for AYAs. This is the first study of a Japanese-language ePRO tool for AYAs that focuses on evaluating supportive care needs, and it should assist both clinicians and researchers in Japan. Our findings may enhance the effectiveness of distress management among AYAs and contribute to AYA support team activities and consistent provision of AYA support.

Furthermore, advanced care planning (ACP) is difficult to implement in AYAs because they have various unmet needs that are generation specific,^1–7^ and it is difficult to determine a proxy decision maker when there is no legal spouse. This ePRO tool may help identify AYAs' concerns and promote ACP. However, there are controversies and concerns about the use of such tools for ACP, and their introduction into clinical setting should be carefully considered.^37,38^

## Conclusion

This study suggests the feasibility of an ePRO tool based on the DTPL-J for AYAs. More research should assess the measurement equivalence between electronic and paper-based PRO measures, sensitivity of the tool to change, and the applicability of the ePRO tool in other hospitals.
